# Coaxial Microincision Cataract Surgery versus Standard Coaxial Small-Incision Cataract Surgery: A Meta-Analysis of Randomized Controlled Trials

**DOI:** 10.1371/journal.pone.0146676

**Published:** 2016-01-08

**Authors:** Xingchao Shentu, Xin Zhang, Xiajing Tang, Xiaoning Yu

**Affiliations:** Eye Center, Second Affiliated Hospital, School of Medicine, Zhejiang University, Hangzhou, China; Sun Yat-sen University, CHINA

## Abstract

**Background:**

We conducted this meta-analysis to compare the outcomes of coaxial microincision cataract surgery (C-MICS) and standard coaxial small incision cataract surgery (C-SICS).

**Methods:**

The outcomes of randomized controlled trials (RCTs) reporting C-MICS and C-SICS were collected from PubMed, Web of Science, and The Cochrane Library in May 2015. The final meta-analysis was conducted on the following intraoperative and postoperative outcomes: ultrasound time (UST), effective phacoemulsification time (EPT), balanced salt solution use (BSS use), cumulative dissipated energy (CDE), mean surgery time, endothelial cell loss percentage (ECL%), best corrected visual acuity (BCVA), increased central corneal thickness (CCT), laser flare photometry values and surgically induced astigmatism (SIA).

**Results:**

A total of 15 RCTs, involving 1136 eyes, were included in the final meta-analysis. No significant between-group differences were detected in EPT, BSS use, CDE, BCVA, laser flare photometry values or increased CCT. However, the C-MICS group showed less SIA (at postoperative day 7: *p*<0.01; at postoperative day 30 or more: *p*<0.01) and greater ECL% (at postoperative day 60 or more: *p*<0.01), whereas the C-SICS group required a shorter UST (*p*<0.01).

**Conclusions:**

The present meta-analysis suggested that the C-MICS technique was more advantageous than C-SICS in terms of SIA, but C-MICS required a longer UST and induced a higher ECL%. Further studies should be done to confirm our results.

## Introduction

Due to recent improvements in phacoemulsification techniques, advances in surgical instruments, and the advent of the foldable intraocular lens (IOL), the C-MICS technique has gained global popularity among ophthalmologists. Prior to these advances, the C-SICS approach, which required a 2.8 to 3.2 mm incision, was the most widely used surgical approach; however, using the C-MICS technique can reduce the incision to less than 2.2 mm [1–2].

In C-MICS, microcoaxial phacoemulsification, irrigation, aspiration, and phacoemulsification are performed with the same instruments as those used in C-SICS [3]. As a result, C-MICS inherits nearly all the advantages of C-SICS, while making smaller incisions. C-MICS patients can then expect more rapid wound healing, smaller SIAs, better post-operative visual acuity, lower risk of infection, and more stable corneal biomechanics following their procedures [4–6]. However, as with other cataract operations, C-MICS may have some disadvantages. Skeptics argue that smaller incisions may be related to decreased followability of the nucleus, reduced efficacy, limited infusions (due to smaller instrument gauges), and increased wound trauma (caused by the relatively tight incisions) [5,7–9]. Numerous clinical trials have been designed and conducted to determine whether C-MICS has more advantages than C-SICS; however, a consensus has not yet been reached. We conducted this meta-analysis to quantitatively ascertain if a switch from C-SICS to C-MICS is necessary.

## Materials and Methods

This meta-analysis was performed in accordance with the Preferred Reporting Items for Systematic Reviews and Meta-Analyses (PRISMA) statement checklist [10].

### Search Strategy and Study Selection

Systemic literature searches were performed in three databases: PubMed, Web of Science and The Cochrane Library. The search covered studies published until May of 2015. The reference lists of relevant papers were then manually screened by the investigators for pertinent articles missed in the primary searches. Search items included:“coaxial”,“microcoaxial”,“microincision”,“microphacoemulsification”,“cataract surgery”, “effective phacoemulsification time”, “balanced salt solution use”, “cumulative dissipated energy”, “surgery time”, “endothelial cell loss percentage”, “best corrected visual acuity”, “ultrasound time”, “increased central corneal thickness”, “laser flare photometry values” and “surgically induced astigmatism” in various combinations.

Only articles that fulfill all of the following criteria were considered for inclusion in this meta-analysis: (1) original RCTs comparing the outcomes of C-MICS with the ourcomes of C-SICS; (2) subjects with no ocular diseases other than cataracts; and (3) C-MICS incision sizes of less than 2.2 mm. The two investigators, Yu and Zhang, independently performed the initial search in terms of abstracts and titles. They then screened the full text of each potential study according to the inclusion criteria detailed above.

All of the selected RCTs were evaluated according to the Jadad scoring system developed by Crowther et al.[11], and studies scoring three or more points were deemed to be of high quality. No specific language restriction was imposed on the selection of publications.

### Data Extraction

The two investigators independently extracted the data using a standardized data extraction format which include the following intraoperative and postoperative outcomes: EPT, BSS use, CDE, surgery time, ECL%, BCVA, UST, CCT, laser flare photometry values and SIA. Any disagreement was decided by the consensus of the investigators. For studies involving more than one microincision size, we used the smaller size.

### Data Synthesis and Statistical Analysis

All statistical analyses in this meta-analysis were conducted using Stata version 12.0 software. The significance level of the statistics was set to *P*<0.01, except in the case of heterogeneity and meta-regression analyses. The means and standard deviations of continuous outcomes were used to calculate the weighted mean difference (WMD) with a 95% confidence interval (CI). Potential heterogeneities among the included studies were assessed using Cochran's Q statistic and an I^2^ index score, with the significance level set at a *P*-value less than 0.10 or an I^2^ score greater than 50% [12]. When high heterogeneity was detected among the included studies, the random effects model based on the DerSimonian and Laird method was used; otherwise, the fixed-effects model based on the inverse variance method was performed [13]. For studies reporting BCVA via the Snellen system, the method introduced by Chen et al. was used to transfer the data to the logMAR system [14]. The methods described by Chen et al. were utilized to calculate EPT, CDE and ECL% [14–16]. A sensitivity analysis was used to assess the robustness of the meta-analysis results by sequentially omitting individual studies. Egger’s linear regression and Begg’s rank correlation tests were used to evaluate the potential publication bias [17–18].

## Results

### Literature Search Results and Characteristics of Included RCTs

**Fig 1:**
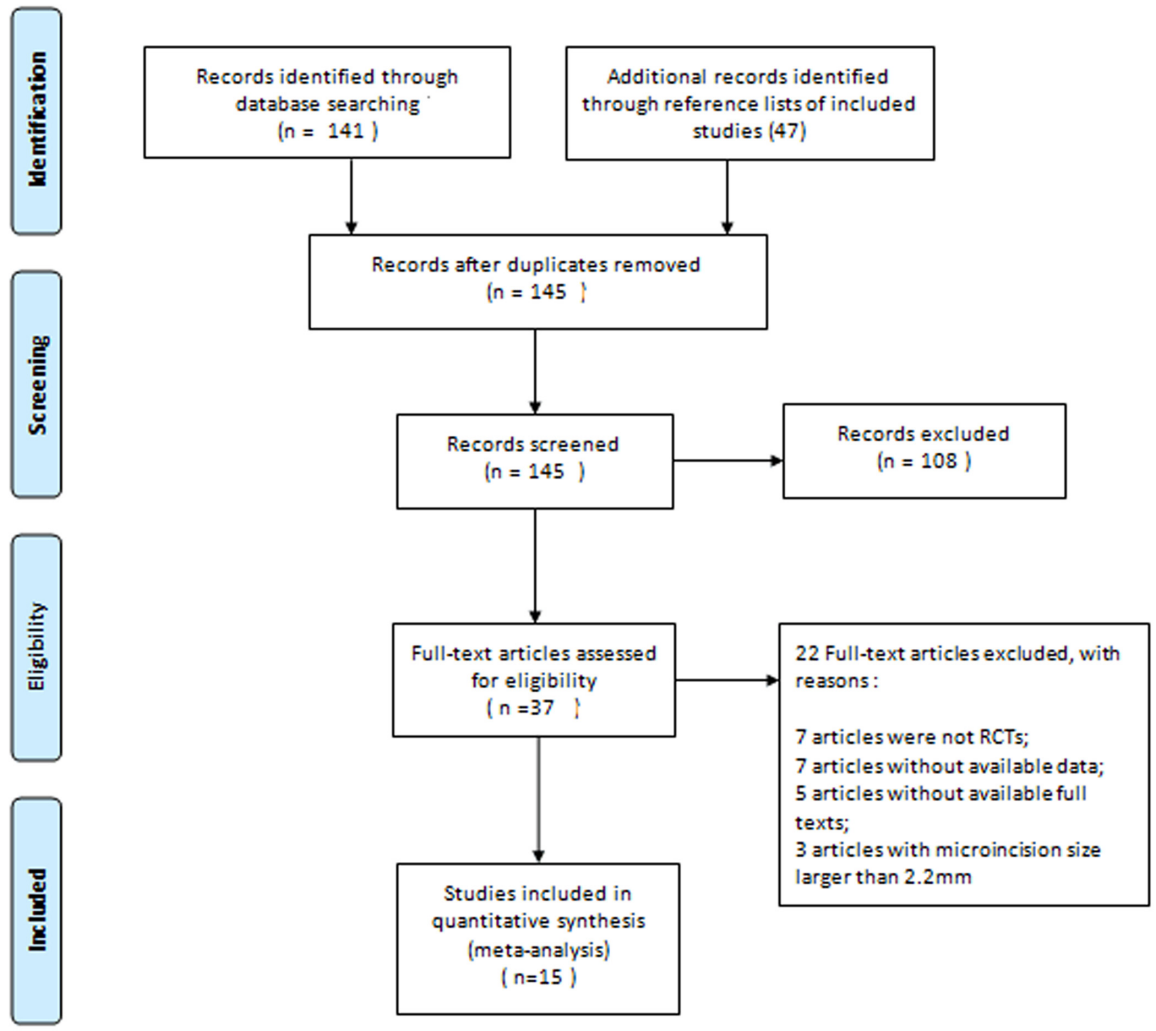
Flow diagram of study selection process.

Of the 145 potentially relevant articles identified by the electronic databases and reference lists of included articles (Fig 1), 37 articles were retrieved for final review after the evaluation of titles and abstracts. Upon further screening, 22 articles were excluded for the following reasons: 7 articles did not involve RCTs [19–25], 7 articles did not provide specific data related to this meta-analysis [26–32], 5 articles were not available in full text [33–37], and 3 articles involved a microincision larger than 2.2 mm [38–40]. Ultimately, 15 studies meeting all predefined inclusion criteria were included in the present meta-analysis [5–7, 41–52].

A total of 1136 eyes were included in this meta-analysis. Of these, 568 were assigned to the C-MICS group, and 568 were assigned to the C-SICS group. The characteristics of the included RCTs were summarized in Table 1. According to the Jadad score, 15 of the included RCTs were considered to be of high quality.

**Table 1:** Characteristics of 15 randomized controlled trials Included into Present Meta-Analysis.

Source(Published Year, Country)	Age (year)	Gender	Sample size	Incision size	Follow-up (day)	Jadad Score
	C-MICS/SICS	M/F	C-MICS/C-SICS	C-MICS/C-SICS		
Can et al.(2010, Turkey)	65.8±13.2/66.2±12.6	36/28	45/45	2.2/2.8	90	1+0–1+0+0–0+1
Hwang et al.(2015, Korea)	64.52±10.65/65.87±12.91	NA	42/42	2.2/2.75	60	1+0–1+0+0–0+1
Hayashi et al.(2014, Japan)	69.3±5.2/69.3±5.2	10/24	34/34	2/2.65	1	1+1–0+1+1–0+1
Dosso et al. (2008, Switzerland)	60-87/60-89	NA	25/25	1.6/2.8	56	1+0–1+0+0–0+1
Samuel et al. (2009, USA)	NA	NA	22/22	2.2/3	42	1+0–1+0+0–0+1
Musanovic et al. (2012Bosnia)	62.06±10.04/65.13±9.59	NA	30/30	2.2/3	30	1+0–1+0+0–0+1
Yao et al.(2011, China)	72±7/73±7	29/51	45/44	1.8/3	90	1+1–0+1+1–0+1
Luo et al.(2012, China)	73.95±6.05/ 72.48±7.19	40/40	40/40	1.8/3.0	90	1+1–0+1+0–0+1
Kim et al.(2011, Korea)	75.95±9.59/72.56±9.05	NA	20/20	1.8/2.75	60	1+0–1+0+0–0+1
Li et al. (2010, Korea)	66.83±9.51/69.25±8.45	39/32	37/39	2.2/2.8	30	1+0–1+0+0–0+1
Wang et al.(2009, China)	69±9/71±8	28/58	43/44	2.2/3	90	1+0–1+0+0–0+1
Hayashi et al.(2009, Japan)	70.1±6.9	21/39	60/60	2/2.65	56	1+1–0+1+0–0+1
Hayashi et al.(2010, Japan)	69.5±6.5/67.8±6.2	16/68	43/41	2/3	56	1+1–0+1+0–0+1
Hashemi et al. (2010, Iran)	66.5±12.0/67.1±11.1	34/40	37/37	2.2/2.8	90	1+0–0+1+0–0+1
Zhu et al.(2014, China)	39–80/48-83	48/42	45/45	2.2/3	90	1+0–0+0+0–0+1

### Intraoperative outcomes

#### Ultrasound Time

**Fig 2:**
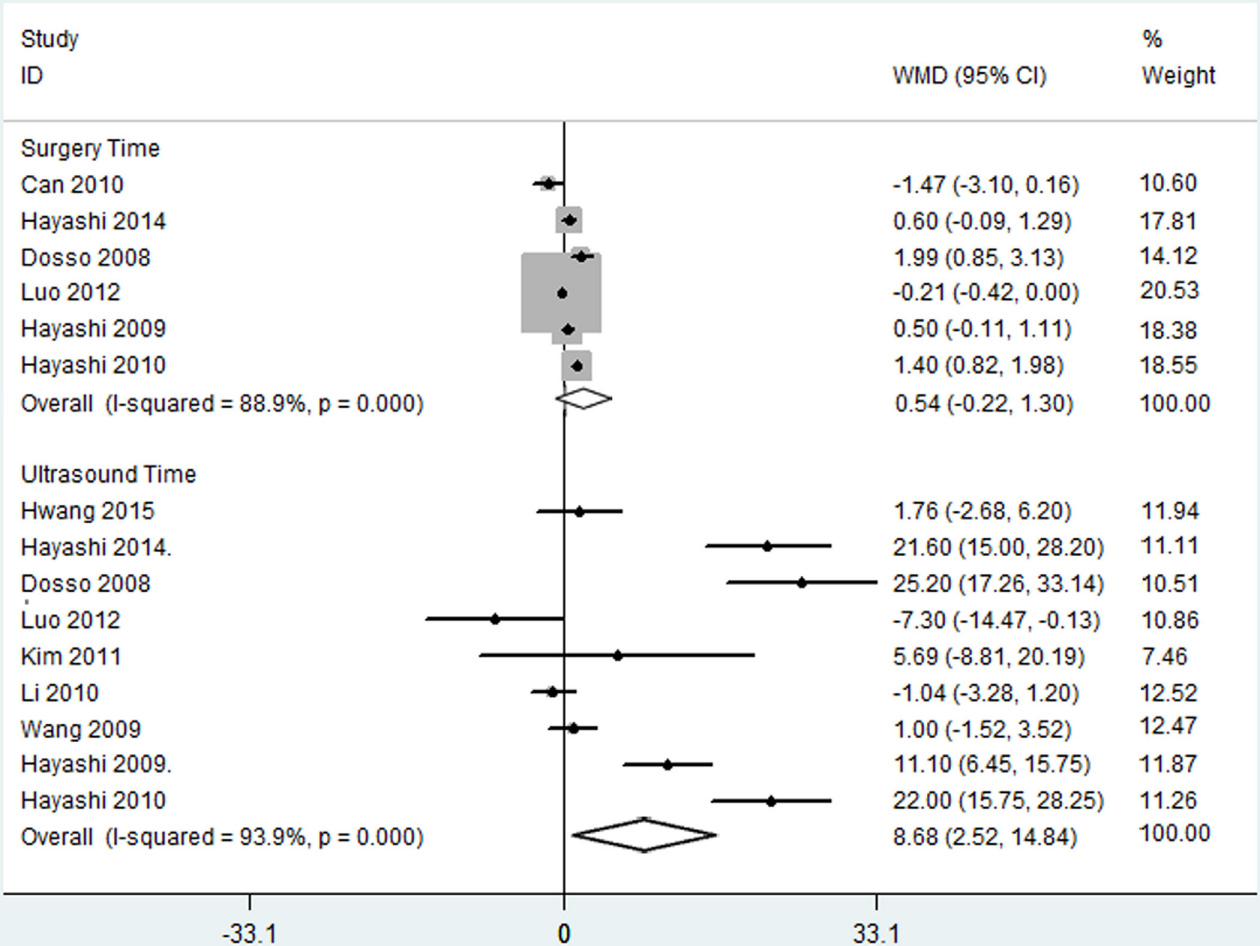
Ultrasound time and surgery time between coaxial microincision cataract surgery and standard coaxial small-incision cataract surgery.

With regard to UST, nine articles yielded information on 689 eyes. According to the forest plot, the C-SICS group required a shorter UST than the C-MICS group in the random effects model (Fig 2, WMD: 8.679, 95% CI: 2.519 to14.839, *p* = 0.006, I^2^ = 93.3%, *P*_heterogeneity_ = 0.0000).

#### Mean Surgery Time

Six RCTs, representing 508 eyes, reported mean surgery time. The pooled results showed no statistical differences between the C-MICS group and the C-SICS group in the random effects model (Fig 2, WMD: 0.541, 95% CI: -0.216 to1.297, *p* = 0.161, I^2^ = 88.9%, *P*_heterogeneity_ = 0.0000)

#### Balanced Saline Use

**Fig 3:**
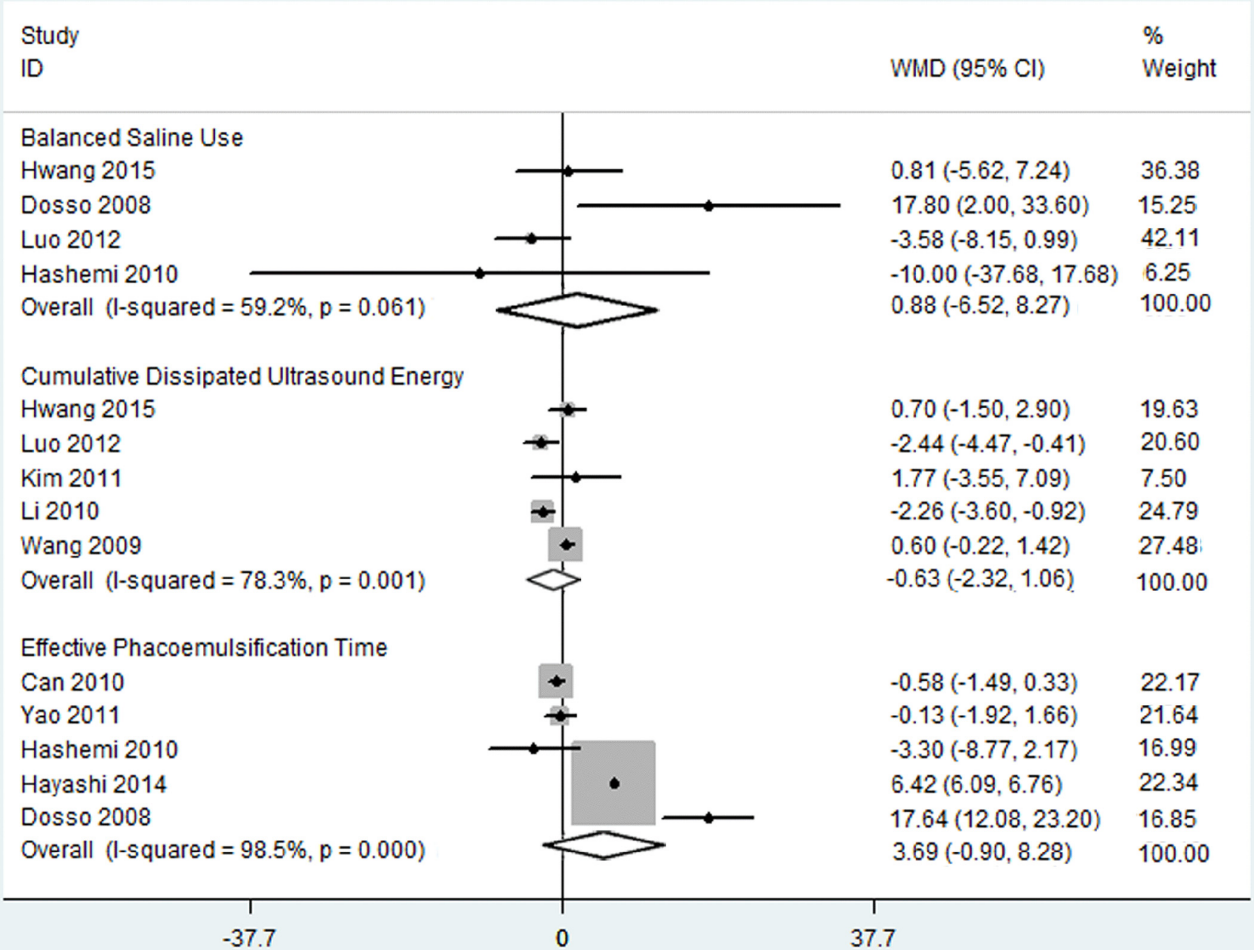
Balanced saline use, cumulative dissipated ultrasound energy, and effective phacoemulsification time between coaxial microincision cataract surgery and standard coaxial small-incision cataract surgery.

Fig 3 showed that four RCTs, representing 288 eyes, were involved in the comparison of the C-MICS and C-SICS groups in the context of BSS use. In the random effects model (I^2^ = 59.2%, *P*_heterogeneity_ = 0.061), no significant between-group differences were detected (WMD: 0.877, 95% CI: -6.521 to 8.274).

#### Effective Phacoemulsification Time

Five RCTs, representing 244 eyes, reported EPT data. Since significant heterogeneity was found among the included RCTs (I^2^ = 98.5%, *P*_heterogeneity_ = 0.000), the random effects model was adopted. Based on the forest plot in Fig 3, no significant difference was found between the C-MICS group and the C-SICS group (Fig 3; WMD: 3.691, 95% CI: -0.904 to 8.284).

#### Cumulative Dissipated Ultrasound Energy

Five studies compared the CDE of the C-MICS group and the C-SICS group, and they involved a total of 362 eyes. Considering the statistically significant heterogeneity (I^2^ = 78.3%, *P*_heterogeneity_ = 0.001), the random effects model was used to reduce errors in this meta-analysis. According to the forest plot in Fig 3, no significant differences were found between the two surgical procedures (WMD: -0.628, 95% CI: -2.317 to 1.061).

### Postoperative Outcomes

#### Postoperative Best Corrected Visual Acuity

Seven articles described postoperative BCVA. We separately conducted subgroup meta-analyses on BCVA at three points in time: within 7 postoperative days, at postoperative day 30 and at postoperatively day 60. Five articles (356 eyes), reported the outcomes of the BCVA within 7 postoperative days, three articles (238 eyes) reported outcomes at day 30, and five articles (378 eyes) reported outcomes at 60 days.

**Fig 4:**
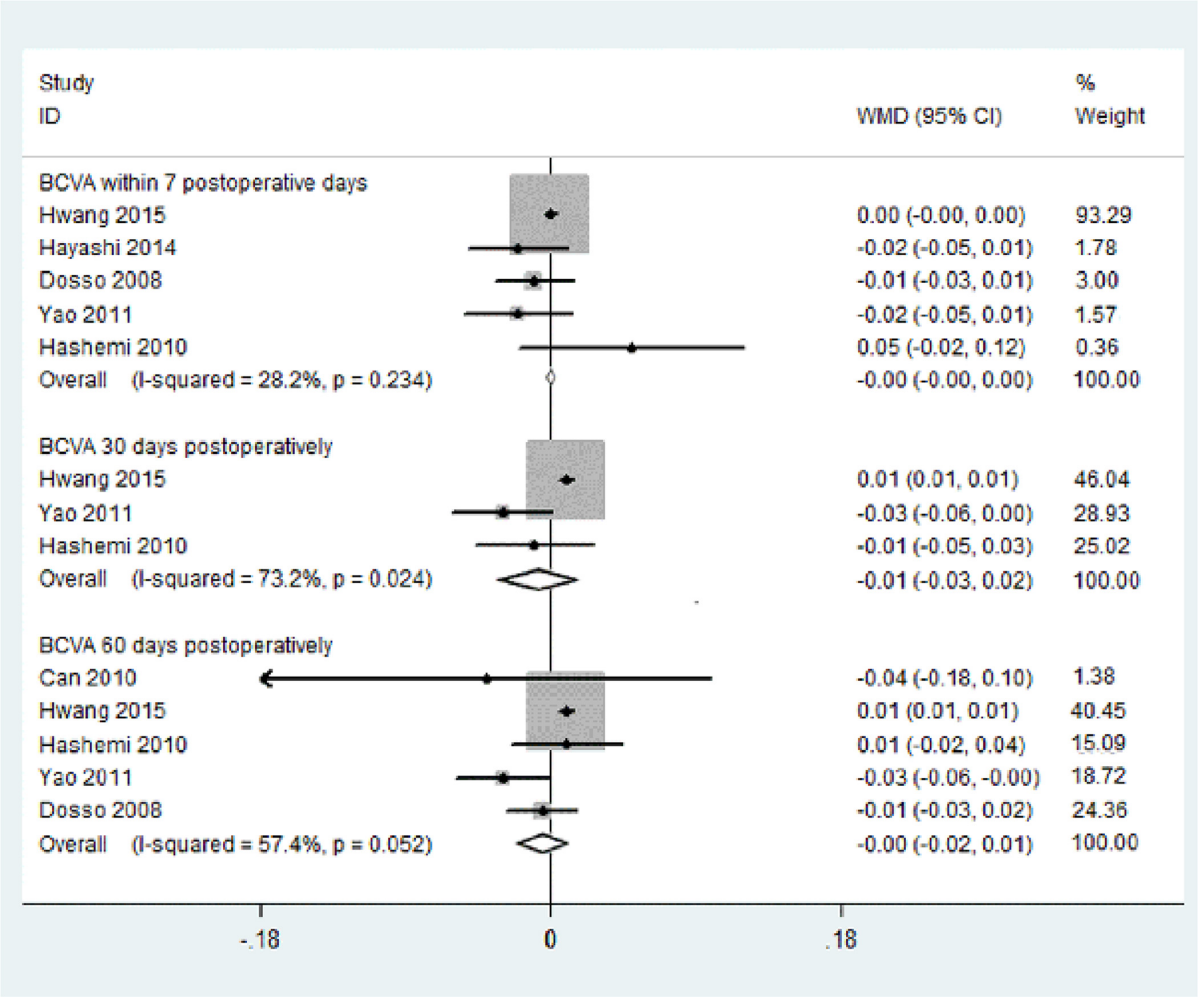
Best corrected visual acuity between coaxial microincision cataract surgery and standard coaxial small-incision cataract surgery.

The pooled results indicated no significant differences between the C-MICS group and the C-SICS group on postoperative BCVA (Fig 4; within 7 days: WMD: -0.001, 95% CI: -0.004 to 0.003, I^2^ = 28.2%, *P*_heterogeneity_ = 0.234; at day 30: WMD: -0.007, 95% CI: -0.033 to 0.020, I^2^ = 73.2%, *P*_heterogeneity_ = 0.024; at day 60: WMD: -0.005, 95% CI: -0.027 to 0.017; I^2^ = 57.4%, *P*_heterogeneity_ = 0.052).

#### Laser Flare Photometry Values

Three studies that were conducted by the same research team reported data for this outcome, and a total of 272 eyes were recruited. No significant differences were found in laser flare photometry values following C-MICS and C-SICS in the fixed effects model (WMD: 0.091, 95% CI: -0.804 to 0.986; I^2^ = 25.6%, *P*_heterogeneity_ = 0.261).

#### Surgically Induced Astigmatism

**Fig 5:**
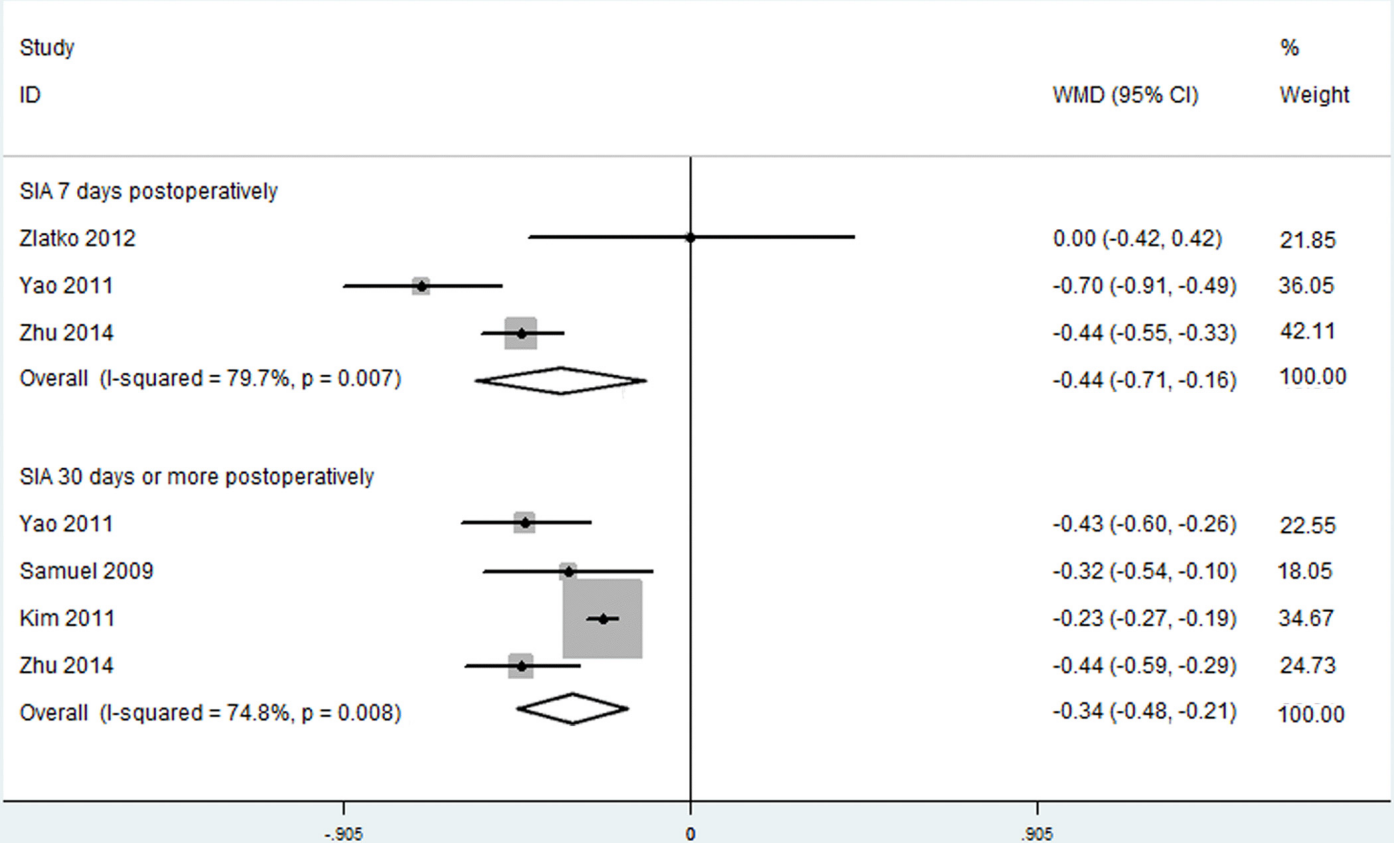
Surgically induced astigmatism between coaxial microincision cataract surgery and standard coaxial small-incision cataract surgery.

We included only SIA data evaluated by vector analysis in this meta-analysis. Three RCTs, representing 230 eyes, compared SIA following C-MICS and C-SICS at 7 postoperative days; and four RCTs, representing 254 eyes, compared SIA at 30 or more postoperative days. The random effects model was applied to reduce errors. The C-MICS group showed less SIAs than the C-SICS group (Fig 5; at day 7: WMD: -0.438, 95% CI: -0.714 to -0.161; at day 30 or more: WMD: -0.343, 95% CI: -0.475 to -0.211).

#### Endothelial Cell Loss Percentage

Six RCTs, involving 354 eyes, reported information concerning ECL% at 7 and 30 postoperative days, while eight RCTs involving 364 eyes reporting the same information at 60 or more postoperative days.

**Fig 6:**
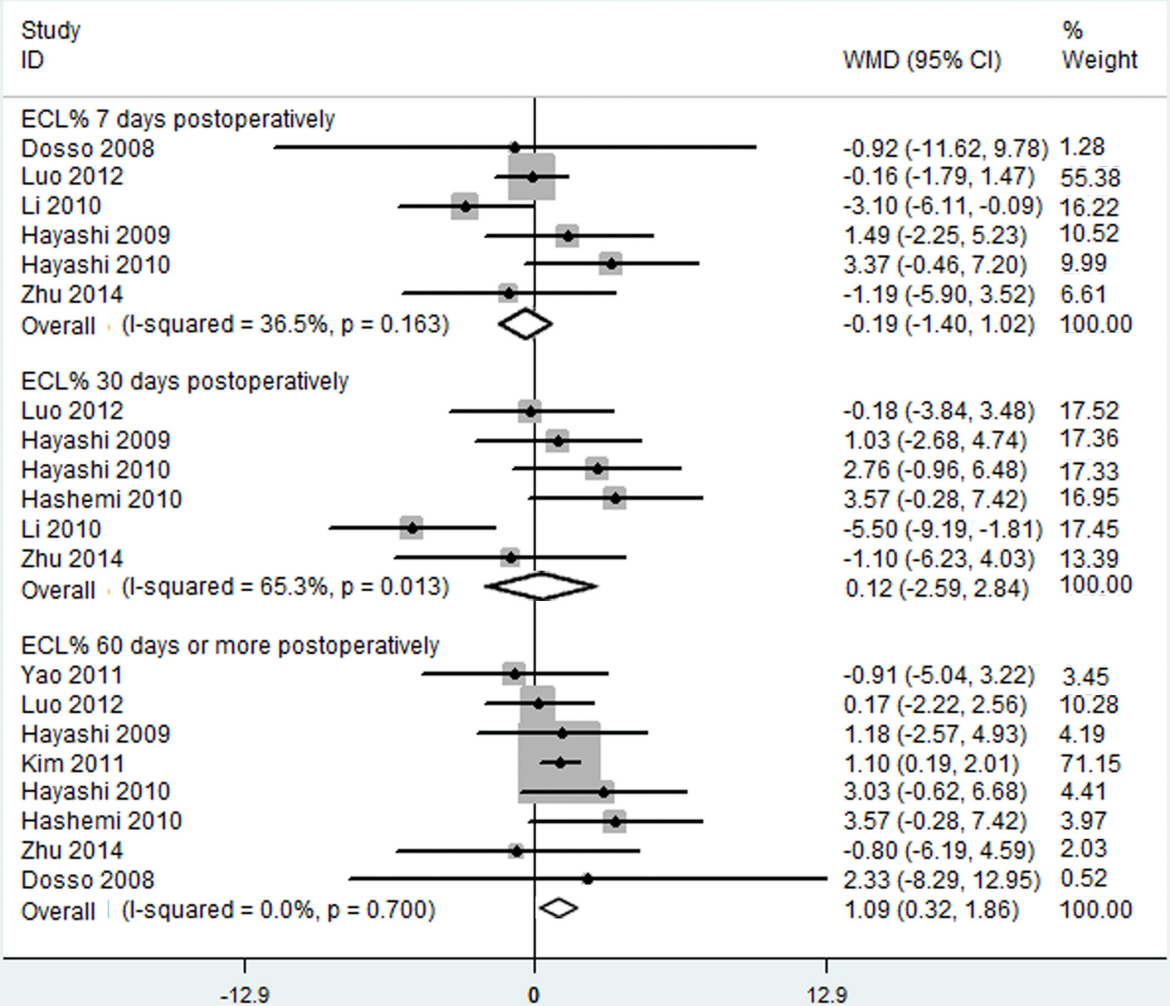
Endothelial cell loss percentage between coaxial microincision cataract surgery and standard coaxial small-incision.

A meta-analysis of the three sets of data indicated no significant between-group differences in ECL% at 1 and 30 postoperative days following C-MICS and C-SICS (at day 7: WMD: 0.-188, 95% CI: -1.400 to 1.023, I^2^ = 36.5%, *P*_heterogeneity_ = 0.163; at day 30: WMD: 0.124, 95% CI: -2.589 to 2.837, I^2^ = 65.3%, *P*_heterogeneity_ = 0.013;), while significant differences at 60 or more postoperative days (Fig 6; WMD: 1.090, 95% CI: 0.322 to 1.857; I^2^ = 0.0%, *P*_heterogeneity_ = 0.700).

#### Increased Central Corneal Thickness

**Fig 7:**
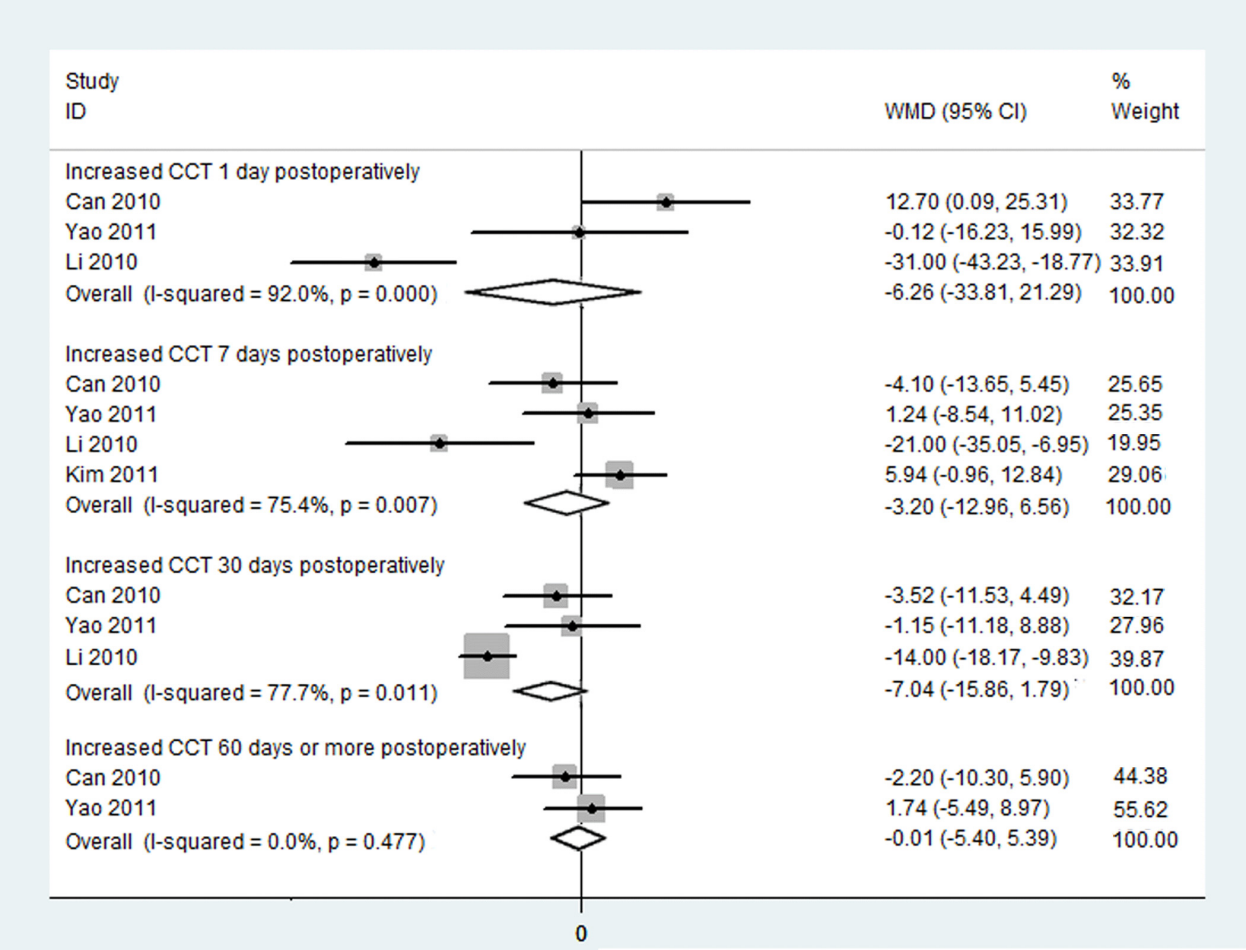
Increased central corneal thickness between coaxial microincision cataract surgery and standard coaxial small-incision cataract surgery.

Four RCTs reported data suggesting increased CCT, and a separate meta-analysis was conducted according to test dates. In the forest plot in Fig 6, there was no significant difference in increased CCT following C-MICS and C-SICS at 1,7,30 and 60 or more postoperative days (Fig 7; at day 1: WMD: -6.261, 95% CI: -33.810 to 21.290, I^2^ = 92%, *P*_heterogeneity_ = 0.000; at day 7: WMD: -3.200, 95% CI: -12.956 to 6.557, I^2^ = 75.4%, *P*_heterogeneity_ = 0.007; at day 30: WMD: -7.036, 95% CI: -15.864 to 1.792, I^2^ = 77.7%, *P*_heterogeneity_ = 0.011; at day 60 or more: WMD: -0.009, 95% CI: -5.404 to 5.387; I^2^ = 0.0%, *P*_heterogeneity_ = 0.477).

### Sensitivity Analysis and Publication Bias

The pooled results, following the sequential omission of individual studies, all fell in the range of the confidence interval with all RCTs included, indicating robust main meta-analysis results.

No publication biases were detected, except an increase in CCT at postoperative day 7 (Begg test: Z = 1.02, *p* = 0.308; Egger test: *p* = 0.048).

### Meta-regression Analyses

We conducted meta-regression analyses to assess the possible effect of different variables in explaining heterogeneity: sample size, study conducted race, publication year and location. The results showed that sample size was the main heterogeneity source of CDE and BCVA at postoperative day 30 (*P*<0.05).

## Discussion

In terms of intraoperative parameters, this systematic meta-analysis involving 15 RCTs revealed no significant differences between the C-MICS group and the C-SICS group with respect to BSS use, CDE, EPT, or mean surgery time; however, the C-MICS group was associated with longer UST. Moreover, in terms of postoperative outcomes, no statistically significant between-group differences were detected in BCVA, CCT increases, or laser flare photometry values. The C-MICS group indicated a higher ECL% and less SIA than the C-SICS group.

With regard to intraoperative parameters, as mentioned above, the only significant difference between the two surgical procedures is the UST. A longer UST requires longer perfusion time under high perfusion pressure but does not imply an increased release of energy in the anterior chamber, given that different machines have different acoustic powers. Moreover, compared to the UST, the EPT and the CDE may serve as better measures of efficiency during surgery, according to the calculation formula. Some researchers have argued that smaller incisions might contribute to lower efficiency, particularly in high-density cataracts [53]. However, in this meta-analysis, the two techniques reported equal efficiency.

The BCVA is considered the most important parameter for evaluating the quality of cataract surgery [16]. Although no differences were detected in the BCVA of the two surgical procedures in our meta-analysis, the C-SICS reported a shorter mean recovery time [5].

Incision size was the main predictor of SIA. Smaller incisions not only induced less SIA, but also led to earlier refraction stabilization and corrected pre-surgical corneal astigmatisms [54–57]. Quantitatively, a corneal astigmatism of 0.5D is considered to be in the upper limit for successful functional visual acuity following IOL implantation [43], given that a 0.5-mm increase in incision size leads to a 0.25D decrease in corneal curvature [58]. Hence, achieving less SIA is an important clinical target for cataract surgery. Our results indicate that the C-MICS technique offers an obvious advantage over the C-SICS technique with respect to SIA.

The postoperative ECL percentage must be a safety consideration in cataract surgery. Moreover, the complete endothelial cell loss process induced by phacoemulsification may last for 10 or more years or after surgery [59]. According to previous studies, the degree of endothelial damage may depend on the surgical technique, type of implanted IOL, emulsification time, age of the patient, cataract density, chattering of the nuclear pieces, corneal manipulation, and amount and type of fluid circulating in the anterior chamber [16, 60–65]. In our study, there were no between-group differences in ECL percentages at postoperative days 7 and 30. However, at postoperative day 60 or later, the C-MICS group exhibited more damage to the endothelial cells, and the discrepancy can be explained by Luo et al. who suggested that C-MICS caused more damage to the integrity of clear corneal incision (such as descemet’s membrane detachment, misaligned incisions, and wound gaps) than C-SICS [6,49]. Compared to C-SICS, the C-MICS’s smaller incision resulted in smaller attracting forces, which tended to increase turbulence [5]. In addition, inflammatory cytokines, such as IL-1b, IL-6, VEGF and PGE2, had higher postoperative expression ratios in the C-MICS group than in the C-SICS group, which indicates that the latter had more sever blood-aqueous barrier breakdowns [41]. Osher et al. reported that smaller incision sizes were related to greater incision temperatures; therefore, the thermal damage that occurs during C-MICS results in more damage to the ECD. Furthermore, according to our results as indicated above, the C-MICS group showed longer UST when compared to the C-SICS group, which means that the former is related to longer perfusion time in the case of high intraocular pressure [66]. Therefore, it is assumed that the C-MICS group brings the possibility of inducing the subsequent corneal endothelial cell loss [67].Considering this, C-MICS is more than C-MICS and therefore a consideration in cataract surgery safety.

No significant differences were detected between the two procedures regarding increases in CCT or laser flare photometry values, which were considered important markers of surgery trauma. Although Hwang et al. reported that inflammatory cytokines in the C-MICS group had higher postoperative expression ratios than in the C-SICS group [41], sub-clinical changes may not build up to measurable levels. What’s more, given the low number of studies included in our study, the results must therefore be interpreted with caution.

The limitations of our meta-analysis should be addressed. First, obvious heterogeneity was detected due to the diversity of the involved patients (e.g., different ages and genders), the surgery programs (e.g., different surgical skills and equipment), and research designs (e.g., different follow-up periods and sample sizes), all of which affected the uniformity of the involved RCTs. Second, we cannot be sure if there were any significant differences in terms of LOCS levels. Specifically, according to Lee et al [53], varying-density cataracts would lead to varying preferences regarding surgical procedures. Third, a publication bias must be taken into consideration, since studies without statistically significant results would not be published. Fourth, for studies involving more than one microincision size, we chose the smaller-sized miroincision, and that, may exaggerate the outcome differences of outcomes between the two groups. Fifth, only one included article reported data about postoperative complications, and the results implied no significant differences between the two groups; thus, we didn’t evaluate the safety of the two types of surgical procedures. Lastly, the studies included in our meta-analysis were too few to improve the accuracy of Egger’s linear regression test or Begg’s rank correlation test in terms of publication bias.

In conclusion, the present meta-analysis showed that the two procedures were not only similar in terms of intraoperative parameters, but also similar in terms of postoperative BCVA, CCT increases, and anterior chamber inflammation levels. Switching from C-SICS to C-MICS is reasonable with regard to seeking less SIA. However, C-MICS was related to a higher ECL percentage, suggesting that surgeons should be cautious when treating patients with corneal endothelial decomposition. Nevertheless, it is notable that no new surgical technique is perfect at the beginning of its development. We believe that, with the development of cryo-phaco techniques, liquid fluid systems and perfusion systems (which should focus on reducing perfusion pressure and perfusion time, increasing phaco efficiency and reducing endothelial cell loss), the future trend will be toward C-SICS. Further studies should be performed to confirm our results and to explore and compare other outcomes of C-MICS versus C-SICS, such as intraoperative and postoperative complications.

## Supporting Information

S1 PRISMA Checklist PRISMA 2009 checklist in this meta-analysis. (DOC)

S1 File The Jadad Score for Assessing the Quality of Studies Included into Present Meta-Analyses. (DOCX)
