# Supplementary material for: Coaxial Microincision Cataract Surgery versus Standard Coaxial Small-Incision Cataract Surgery: A Meta-Analysis of Randomized Controlled Trials
Source: PLoS One. 2016 Jan 8;11(1):e0146676. doi: 10.1371/journal.pone.0146676 (PMC4706354; doi:10.1371/journal.pone.0146676)
Supplement: S1 File — (DOCX) [file pone.0146676.s002.docx]

**The Jadad Score for Assessing the Quality of Studies Included into Present Meta-Analyses**
Question 1. Was the study described as randomized? If yes, score 1 point.
Question 2. If yes to question 1, was an appropriate randomization sequence described and used (e.g., table of random numbers, computer generated)? If yes, score 1 point.
Question 3. If yes to question 1, was an inappropriate method to generate the sequence of randomization used (e.g., patients were allocated alternately, or according to date of birth, hospital number)? If yes, subtract 1 point.
Question 4. Was the study described as double-blinded? If yes, score 1 point.
Question 5. If yes to question 4, was an appropriate method of blinding used? If yes, score 1 point.
Question 6. If yes to question 4, was an inappropriate method for blinding used? If yes, subtract 1 point.
Question 7. Were the withdrawals and dropouts described? If yes, score 1 point.
